# Frailty Scores and Ethical Decision‐Making in Critical Care: Lessons From the COVID‐19 Pandemic

**DOI:** 10.1002/ccr3.73448

**Published:** 2026-09-02

**Authors:** Chukwuka Elendu, Chidera A. Okezie‐Okoye, Ahmed I. Ali, Emeka H. Okolo, Mirabel A. Bell‐Gam, Kiranraj Ravikumar, Favour K. Mbakwe

**Affiliations:** ^1^ Federal University Teaching Hospital Owerri Nigeria; ^2^ Federal Medical Center, Jabi Abuja Nigeria; ^3^ Medical Academy Named After S.I. Georgievsky of Vernadsky Simferopol Russia; ^4^ The Park Hospital Nottingham UK; ^5^ CUNY Bronx Community College Bronx New York USA; ^6^ Belarusian State Medical University Minsk Belarus; ^7^ Obafemi Awolowo University Teaching Hospitals Complex Ile‐Ife Nigeria

## Abstract

Frailty scores should support, not replace, individualized critical care decisions, as the COVID‐19 pandemic exposed the ethical limitations of rigid frailty‐based triage, particularly among older adults and individuals with chronic disability. Meaningful recovery may occur despite severe illness, reinforcing the importance of patient autonomy, multidisciplinary assessment, and holistic clinical judgment.

Dear Editor,

We read with great interest the recent report describing the ethical and clinical challenges associated with frailty‐based triage during the COVID‐19 pandemic [1].

The COVID‐19 pandemic placed healthcare systems under unprecedented strain, forcing difficult decisions regarding intensive care admission and other life‐sustaining therapies. During the early phases of the pandemic, many institutions adopted triage strategies based on age, comorbidity burden, and prognostic scoring systems to maximize survival amid resource scarcity. Although these approaches emerged from legitimate public health concerns, they also exposed the limitations of reductionist decision‐making models that risked conflating chronological age with biological vulnerability and frailty with futility [1, 2].

Frailty has become an important construct in critical care because it reflects diminished physiological reserve and vulnerability to stressors. During the pandemic, the Clinical Frailty Scale (CFS) gained widespread use because of its simplicity and rapid bedside applicability [3]. Observational studies associated higher frailty scores with increased mortality among hospitalized COVID‐19 patients [4, 5], leading some national guidelines to incorporate frailty assessment into triage algorithms for ICU admission and escalation of care. However, prognostic association does not equate to deterministic prediction, as frailty scores estimate population‐level probabilities rather than individual outcomes. The concern arises when such tools are used as rigid gatekeeping instruments that overlook the heterogeneity of aging and the multidimensional nature of resilience.

The described patient exemplifies the complexity of frailty‐based decision‐making because, despite severe acute illness and multiple comorbidities, he maintained meaningful social engagement, preserved quality of life, and clearly expressed wishes for full intensive treatment. His recovery challenges assumptions that advanced age and elevated frailty scores necessarily predict poor post‐ICU outcomes. The report also highlights an important limitation of frailty instruments: their potential invalidity in individuals with lifelong disabilities or chronic neurological impairments unrelated to age‐associated decline. In such cases, functional dependence may reflect stable disability rather than progressive biological frailty, and failure to distinguish between the two risks reinforcing structural inequities and indirect discrimination against vulnerable populations [1, 6].

Ethical decision‐making in critical care traditionally rests on the principles of beneficence, nonmaleficence, autonomy, and justice. During pandemics, however, crisis standards of care are often shaped by utilitarian efforts to maximize aggregate benefit amid resource shortages. Although such an approach may appear ethically justifiable, excessive reliance on utilitarian frameworks can marginalize individual dignity and undermine trust in healthcare systems [7]. The pandemic particularly heightened concerns among older adults, nursing home residents, and persons with disabilities that they would be deprioritized for life‐sustaining interventions [8].

Early in the pandemic, commentators warned that inappropriate use of frailty scales could create self‐fulfilling prophecies, whereby assumptions of poor prognosis lead to denial of intensive interventions and consequently poorer outcomes that appear to validate the original decision [6]. Such circular reasoning undermines the integrity of prognostic tools and risks normalizing ageism within emergency medicine. In addition, triage decisions made under crisis conditions remain susceptible to implicit bias, emotional exhaustion, institutional pressures, and overreliance on simplified scoring systems at the expense of individualized deliberation.

Patient autonomy remains a central consideration in this context, particularly as critically ill older adults are frequently excluded from meaningful goals‐of‐care discussions and advance care planning remains underutilized globally [9, 10]. Advanced age alone should not be interpreted as a preference for treatment limitation, as many elderly individuals prioritize survival, meaningful interaction, and preservation of personal identity despite anticipated functional impairment. In the report, the patient clearly desired aggressive treatment, with full family support for these wishes [1]. Respecting autonomy in such situations requires avoidance of paternalistic assumptions regarding acceptable quality of life, particularly given the documented discordance between clinicians' perceptions of acceptable outcomes and patients' valuations of life with disability or dependency [11].

The pandemic also renewed attention to the distinction between biological and chronological age, particularly because chronological age remains an imperfect surrogate for physiological reserve. Older adults represent a highly heterogeneous population with substantial variation in cognition, mobility, immune competence, and recovery potential. Consequently, age thresholds alone are ethically and scientifically indefensible as exclusion criteria for intensive care, especially as some nonagenarians maintain high functional independence while younger individuals may exhibit profound frailty due to chronic disease or socioeconomic disadvantage. Contemporary geriatric medicine therefore emphasizes individualized assessment incorporating cognition, mobility, nutritional status, social support, comorbidity burden, and patient priorities rather than simplistic age‐based categorization [12].

Nevertheless, frailty assessment retains important clinical utility when appropriately contextualized, as it identifies patients at increased risk for delirium, prolonged hospitalization, institutionalization, and mortality [13, 14]. It may also facilitate anticipatory care planning, early rehabilitation, nutritional optimization, multidisciplinary interventions, and improved communication regarding prognosis and treatment goals in perioperative and critical care settings. The concern therefore lies not in frailty measures themselves, but in their use as isolated determinants of access to care, as prognostic tools should inform rather than replace clinical judgment.

The patient's recovery trajectory further highlights the adaptability of some elderly individuals following severe critical illness. Despite prolonged ventilation, bacterial superinfection, pneumothorax, dialysis‐dependent renal dysfunction, and extensive pulmonary involvement (Figure 1), he regained substantial baseline function and returned to his residential facility [1]. This outcome reinforces evidence that meaningful recovery after intensive care remains possible even among very old adults, many of whom report acceptable quality of life months to years after ICU discharge [15, 16]. Importantly, perceptions of quality of life are highly subjective and cannot be reliably predicted solely by demographic characteristics or acute illness severity scores.

**FIGURE 1:**
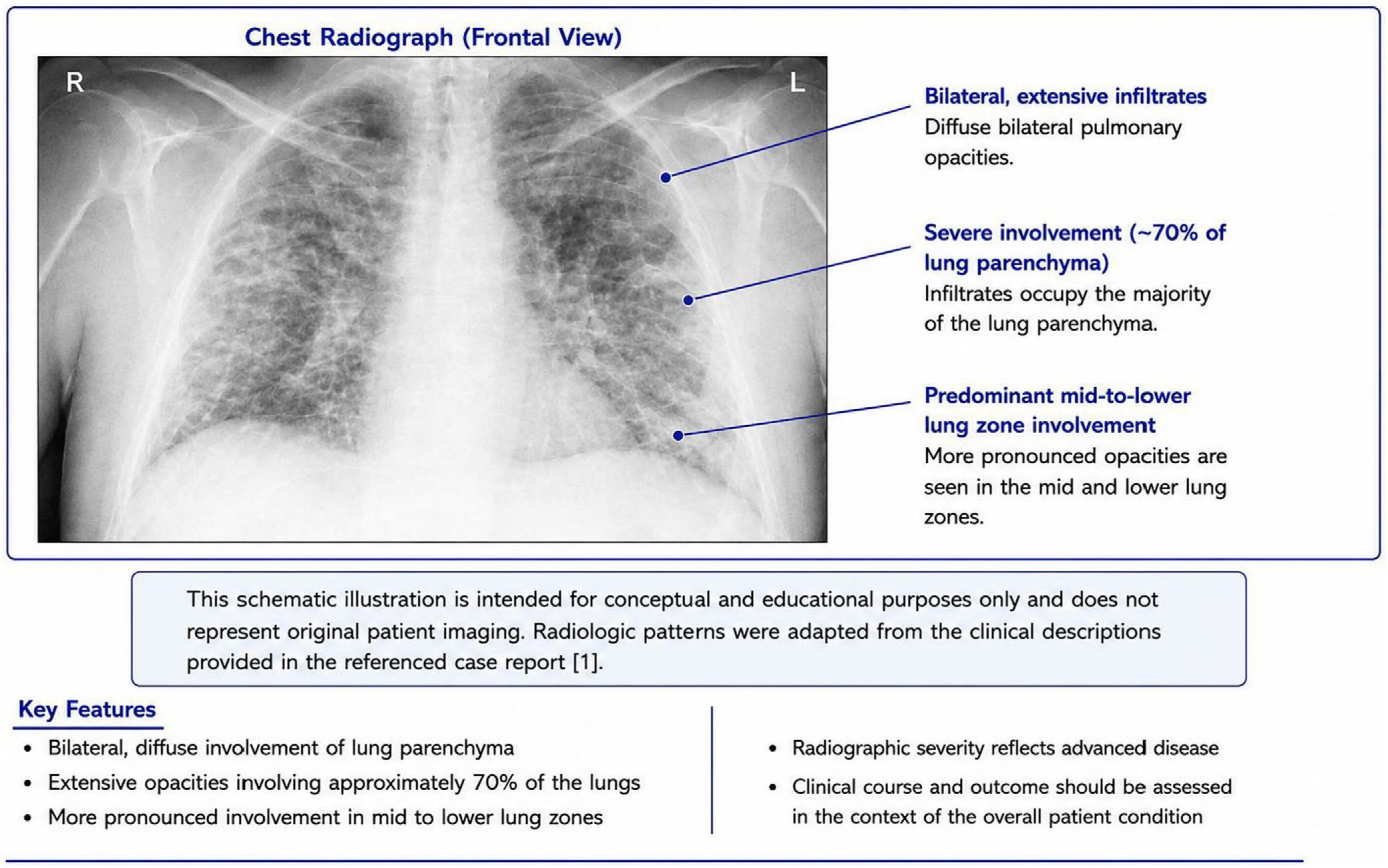
Schematic chest radiograph illustrating diffuse bilateral pulmonary infiltrates described in severe COVID‐19 pneumonia.

The report also raises broader societal concerns regarding the valuation of elderly lives during public health emergencies, particularly as public discourse during the pandemic occasionally portrayed older adults as expendable or inevitable casualties of crisis conditions. Such narratives risk weakening commitments to equitable care and intergenerational solidarity, underscoring the need for disaster medicine frameworks that avoid implying that advanced age diminishes moral worth. Instead, every patient deserves individualized consideration grounded in clinical evidence, ethical consistency, and human dignity [1].

In addition, the patient's experience carries particular historical significance because of his childhood exposure to threats associated with Nazi‐era eugenic practices [1]. Although contemporary triage protocols are fundamentally distinct from such historical atrocities, the symbolic implications of denying care to elderly or disabled individuals cannot be ignored. Modern medical ethics have evolved to prevent discriminatory valuation of human life based on disability, productivity, or perceived social utility, and this historical context should encourage humility and caution when developing crisis allocation frameworks.

Another lesson from the pandemic is the importance of transparency in triage policy development. During early COVID‐19 surges, unclear and rapidly evolving allocation guidance contributed to clinician moral distress, emotional burnout, and public anxiety. Future pandemic preparedness should therefore prioritize transparent, multidisciplinary triage frameworks developed collaboratively by intensivists, geriatricians, ethicists, legal scholars, disability advocates, and community representatives, with emphasis on procedural fairness, consistency, accountability, and mechanisms for appeal or reassessment (Figure 2) [17].

**FIGURE 2:**
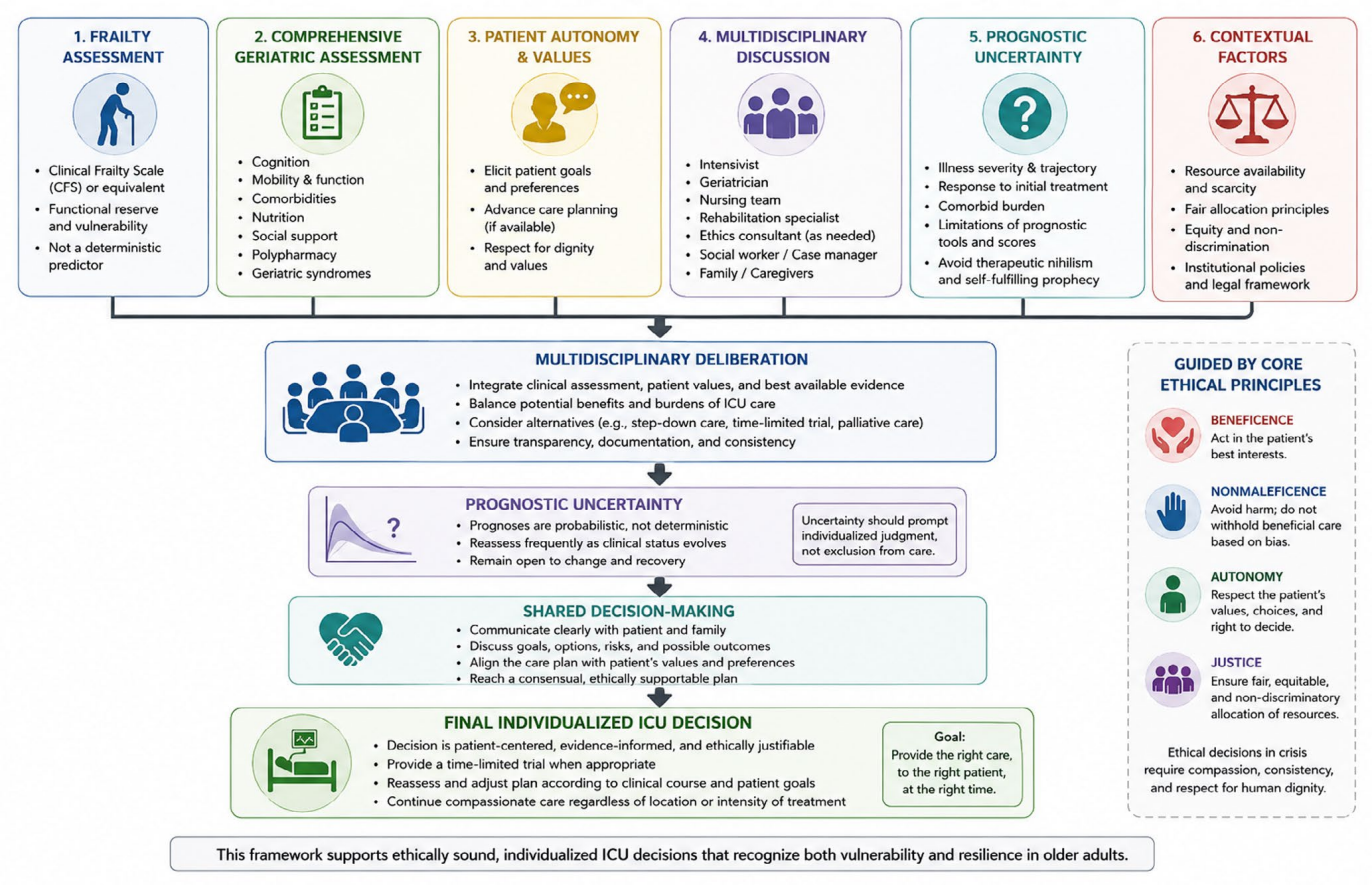
Ethical framework for individualized critical care decision‐making in frail older adults during healthcare crises.

Furthermore, prognostic modeling should move beyond reliance on static scoring systems toward more dynamic and individualized approaches. Machine learning, biomarker integration, longitudinal frailty trajectories, and multidimensional geriatric assessments may improve prognostic precision in critically ill older adults. However, even sophisticated predictive models cannot fully capture human resilience, psychosocial adaptation, or personal values, and clinical uncertainty remains inherent to critical care medicine. Ethical practice therefore requires individualized judgment and compassionate flexibility rather than rigid algorithmic determinism.

The pandemic also exposed disparities in healthcare access affecting socioeconomically disadvantaged populations, racial minorities, and residents of long‐term care facilities. Frailty‐based triage frameworks may inadvertently amplify these inequities because frailty is strongly shaped by social determinants of health, including poverty, malnutrition, chronic stress, and inadequate healthcare access [18]. Consequently, prioritization systems that heavily weight frailty risk perpetuating structural injustice under the guise of objective clinical assessment. Ethical allocation models must therefore account for social vulnerability and avoid reinforcing preexisting inequities.

Communication with patients and families remained an equally important challenge during the pandemic, particularly as discussions regarding prognosis, intensive interventions, and treatment limitations became more difficult because of visitation restrictions, emotional distress, and rapidly evolving clinical conditions. Compassionate communication therefore remains central to ethical critical care, particularly as prognostic estimates are inherently probabilistic and uncertainty persists even in apparently high‐risk cases. Honest discussions of risks and potential outcomes should coexist with respect for hope and patient‐defined goals.

The presented case also underscores the value of interdisciplinary collaboration, as ethical decision‐making in critical care should not rest solely on individual clinicians operating under extreme pressure [1]. Multidisciplinary involvement from intensivists, geriatricians, nurses, rehabilitation specialists, ethicists, and family representatives may reduce bias and support more balanced decisions. Nurses, in particular, provide important insights into patient values, functional status, and psychosocial context, which are essential when evaluating treatment proportionality and long‐term recovery potential.

From a policy perspective, the pandemic demonstrated that resource scarcity is partly a systems‐level failure rather than solely an unavoidable consequence of crisis conditions. Chronic underinvestment in healthcare infrastructure, critical care capacity, workforce resilience, and long‐term care preparedness substantially contributed to triage pressures in many countries. Ethical analysis should therefore extend beyond bedside allocation decisions to encompass societal obligations regarding healthcare preparedness, equitable resource distribution, and sustained investment in surge capacity, staffing support, public health infrastructure, and geriatric care systems.

Importantly, the case should not be interpreted as evidence that all elderly or frail patients benefit from maximal intervention irrespective of prognosis, but rather as a reminder of the dangers of premature therapeutic nihilism [1]. Some patients may appropriately choose comfort‐focused care, and in certain situations aggressive intervention may prove nonbeneficial or burdensome. Ethical practice therefore depends on individualized assessment rather than categorical inclusion or exclusion, requiring clinicians to navigate uncertainty while honoring patient values and avoiding discriminatory assumptions.

The long‐term implications of the pandemic for geriatric critical care will likely persist beyond acute viral surges. Frailty assessment will remain integral across perioperative medicine, emergency care, oncology, nephrology, and intensive care but should serve as one component of holistic clinical reasoning rather than a standalone determinant of treatment eligibility. Comprehensive geriatric assessment, shared decision‐making, and continuous reassessment of therapeutic response should therefore remain central to critical care ethics.

In conclusion, frailty scores should function as adjunctive clinical tools rather than definitive determinants of intensive care eligibility. The COVID‐19 pandemic highlighted both the necessity and limitations of triage systems, reinforcing that prognostic tools should support rather than replace individualized care, particularly as meaningful recovery may still occur even in seemingly unfavorable circumstances.

## Author Contributions

**Chukwuka Elendu:** conceptualization, investigation, data curation, methodology, formal analysis, writing – review and editing, writing – original draft, visualization, validation, supervision, project administration. **Chidera A. Okezie‐Okoye:** investigation, validation, writing – original draft. **Ahmed I. Ali:** writing – review and editing, validation. **Emeka H. Okolo:** writing – review and editing, visualization, validation. **Mirabel A. Bell‐Gam:** validation, writing – review and editing, investigation. **Kiranraj Ravikumar:** validation, writing – review and editing. **Favour K. Mbakwe:** validation, writing – review and editing.

## Funding

The authors have nothing to report.

## Disclosure

The views expressed in this report are solely those of the authors and do not represent the official positions of any affiliated institutions.

## Ethics Statement

Ethical approval was not required, as this letter does not involve new patient data, human subject research, or original clinical investigation.

## Consent

Written informed consent for publication was obtained where applicable. This letter does not include new patient data.

## Conflicts of Interest

The authors declare no conflicts of interest.

## Data Availability

Data sharing not applicable to this article as no datasets were generated or analysed during the current study.
